# Toxicity and Influence of Sublethal Exposure to Sulfoxaflor on the Aphidophagous Predator *Hippodamia variegata* (Coleoptera: Coccinellidae)

**DOI:** 10.3390/toxics11060533

**Published:** 2023-06-14

**Authors:** Panagiotis J. Skouras, Eirini Karanastasi, Vasilis Demopoulos, Marina Mprokaki, George J. Stathas, John T. Margaritopoulos

**Affiliations:** 1 Laboratory of Agricultural Entomology and Zoology, Department of Agriculture, Kalamata Campus, University of the Peloponnese, 24100 Antikalamos, Greece; marina.brokaki.97@gmail.com (M.M.); g.stathas@uop.gr (G.J.S.); 2 Laboratory of Plant Protection, Department of Agriculture, Kalamata Campus, University of the Peloponnese, 24100 Antikalamos, Greece; v.dimopoulos@go.uop.gr; 3 Plant Protection Laboratory, Department of Agriculture, University of Patras, 30200 Messolonghi, Greece; ekaranastasi@upatras.gr; 4 Department of Plant Protection, Institute of Industrial and Fodder Crops, Hellenic Agricultural Organization “DEMETER”, 38334 Volos, Greece; johnmargaritopoulos@elgo.com

**Keywords:** biological control, ecotoxicology, insecticides, IPM, side effect, coccinellids

## Abstract

*Hippodamia variegata* (Goeze), the variegated ladybug, is a predator of many insect pests, especially aphids. Sulfoxaflor is a chemical insecticide that can be used to control many sap-feeding insect pests, for instance, plant bugs and aphids, as an alternative to neonicotinoids in different crops. To improve the combination of the *H. variegata* and sulfoxaflor in an IPM (integrated pest management) program, we studied the ecological toxicity of the insecticide to the coccinellid predator at sublethal and lethal doses. We examined the influence of sulfoxaflor on larvae of *H. variegata* using exposure doses of 3, 6, 12, 24, 48 (maximum recommended field rate (MRFR)), and 96 ng a.i. per insect. In a 15-day toxicity test, we observed decreased adult emergence percentage and survival, as well as an increased hazard quotient. The LD_50_ (dose causing 50% mortality) of *H. variegata* due to sulfoxaflor decreased from 97.03 to 35.97 ng a.i. per insect. The total effect assessment indicated that sulfoxaflor could be grouped as slightly harmful for *H. variegata*. Additionally, most of the life table parameters were significantly decreased after exposure to sulfoxaflor. Overall, the results present a negative influence of sulfoxaflor on *H. variegata* when applied at the recommended field dose for controlling aphids in Greece, which demonstrates that this insecticide may only be employed with care when used in IPM programs.

## 1. Introduction

The green peach-potato aphid, *Myzus persicae* (Sulzer) (Hemiptera Aphididae), is one of the main pests in peach orchards and various herbaceous crops in Greece and worldwide [1]. It is a polyphagous pest that infests over 400 plant species belonging to 40 distinct plant families [2].

*Myzus persicae* control relies on the use of chemical insecticides. However worldwide, the species has developed resistance to several classes of insecticides over the years, such as carbamates, organophosphates, neonicotinoids, and pyrethroids [3,4,5]. To date, at least seven mechanisms have been described regarding resistance to 84 active ingredients [6]. For example, populations collected in China have developed 5.6–115.0-fold resistance to thiacloprid, nitenpyram, chlorpyrifos, thiamethoxam, cyantraniliprole, and clothianidin compared to the susceptible populations [7] (for comprehensive reviews on this topic, see [3,4,5,6]). Furthermore, the extensive use of insecticides for their control of adversity affects many natural enemies (predators and parasites) of plant pests, including those of *M. persicae* [8,9,10,11], as well as the environment [12]. To overcome or delay the development of pest insecticide resistance, several strategies are available, such as insecticide resistance monitoring, alternation of active ingredients with different modes of action, and the incorporation of different control tools in Integrated Pest Management (IPM) schemes. On this basis, the combination of biological control agents (for instance, parasitoids and predators) with insecticides is an environmentally benign approach to manage pests in a socially acceptable and economically viable manner [13].

In various countries, the predatory ladybeetle, *Hippodamia variegata* (Goeze) (Coleoptera: Coccinellidae), is one of the most crucial natural enemies of aphid pests, including *Dysaphis crataegi*, *Aphis fabae* (Hemiptera: Aphididae), and *M. persicae* [14,15,16]. Certain biological traits of *H. variegata,* such as voracity, predation capacity, aphid consumption, and high reproductive rate, are responsible for its efficiency as a biological control agent. These traits have been well studied in different predator–aphid models [14,15,16].

A novel sulfoximine Insecticide, sulfoxaflor (Group 4C), was developed by Dow AgroSciences in 2010 [17]. Sulfoximines may be considered as fourth-generation neonicotinoid insecticides due to their similar mode of action. Nevertheless, sulfoxaflor is a nAChR (nicotinic acetylcholine receptor) competitive agonist/modulator that binds to nAChR in place of acetylcholine in the central nervous system of insects [18], in a manner different to other nAChR acting insecticides and neonicotinoids [15]. Sulfoxaflor is an effective insecticide for the management of piercing and sucking insect pests belonging to many families, such us Aphididae, Miridae, and Aleyrodidae [19].

Within this framework, the purpose of the present study was to investigate the long-term toxicity of sulfoxaflor on *H. variegata*. At this point, we have found the LD_50s_, NOER_S_ (No Observed Effect Application Rates) from chronic exposure for the 2nd instar larvae of *H. variegata* in laboratory microcosms. This work may contribute to optimize the use of sulfoxaflor in IPM programmes, to protect natural enemies, and to maximize control of sap sucking insects. 

## 2. Materials and Methods

### 2.1. Insecticide and Tested Concentrations 

Commercial formulations of sulfoxaflor (Closer 120SC, Dow AgroSciences Greece) were dissolved in HPLC-grade acetone at different concentrations for the trials. The concentrations, applied on the biological control agent *H. variegata*, (3, 6, 12, 24, 48, and 96 ng a.i. per insect), were double diluted with acetone. The manufacturer’s maximum recommended field dose for controlling aphids in Greece is 48 ng sulfoxaflor per insect. 

### 2.2. Test Species

#### Hippodamia Variegata

*H. variegata* laboratory colonies were obtained from individuals (approximately 100 adults coccinellids predators) collected in July 2017 from tobacco fields in the Meliki area in northern Greece [8]. The rearing procedure of *H. variegata* was performed according to (Skouras et al., 2019; 2021) [10,11]. Individuals of *H. variegata* were reared in cylindrical acrylic glass cages (50 ht × 30 diam. cm) and maintained in an environmentally controlled room at 25 ± 1 °C, 65 ± 2% relative humidity (RH) and a 16 L:8 D photoperiod. *H. variegata* was reared on *A. fabae* and *M. persicae*, which were maintained on *Vicia faba* (broad beans) at 20 ± 1 °C, 50 ± 5% RH, and 16 L:8 D. 

### 2.3. Bioassays

#### Biological Control Agent Bioassays 

The lethal toxicity and effect of the six concentrations of sulfoxaflor on *H. variegata* were studied in the laboratory by exposing second instar larvae (between 12–24 h old) to the insecticide through topical application, using a 10 μL Hamilton microsyringe [10,11]. The insecticide solution was applied in 1 μL of acetone to the mesonotum of each larva. Controls were treated with acetone only. Twenty larvae were examined for each sulfoxaflor concentration or control, and three replications per treatment were performed. The criterion for death was the failure of the insects to move their legs when stimulated with a fine brush. Larvae mortality, duration of the different life stages, pupae formation, and successful adult emergence were scored. Additionally, sex ratio, male and female adult longevity, fresh mass, fecundity, and adult or total pre-oviposition period (APOP and TPOP, respectively) were scored.

### 2.4. Risk Assessment

In order to assess the sulfoxaflor toxicological risk to *H. variegata*, we used the daily hazard quotient (HQ), which was estimated by dividing the maximum field recommended dose of sulfoxaflor by the sulfoxaflor concentration causing 50% mortality (LD_50_) to *H. variegata* obtained from a laboratory study [20]. Ratios equal to or greater than 2 indicate sulfoxaflor as a potential hazard to *H. variegata*. Ratios lower than 2 indicate a reduced intoxication risk. Using the Overmeer and van Zon (1982) formulas [21], the total effect (E) was calculated using the equation.

E (%) = 100 − (100 − M_c_) × ER (1)

where ER is the ratio of the mean weekly number of laid eggs by treated *H. variegata* females versus the number of control females, and M_c_ is the final corrected mortality. The insecticide sulfoxaflor has been classified into four toxicity categories, i.e., 1. harmless (Ε < 30%); 2. slightly harmful (30 ≤ Ε ≤ 79%); 3. moderately harmful (80 ≤ Ε ≤ 99%); and 4. harmful (Ε > 99%), according to the IOBC laboratory scale (International Organisation for Biological Control) [22].

### 2.5. Statistical Analysis 

The mortality–dose relationship, LD_50_, was calculated by probit analysis using SPSS version 26.0 (SPSS Inc., Chicago, IL, USA). The population, life table parameters, and population projection are shown in Supplementary Materials. The developmental duration time and survival rates between the different stage/instars were compared using a repeated measure ANOVA to examine differences amongst the treatment groups. The Kolmogorov-Smirnov test was used to determined data normality. NOER (No Observed Effect Application Rates) values were estimated from the treatment comparison using a one-way ANOVA. All between or among-group differences of means were compared by Tukey’s test (HSD, *p* ≤ 0.05) 

## 3. Results

### 3.1. Toxicity and Influence of Sulfoxaflor on the Survival Rate of H. variegata 

**Figure 1:**
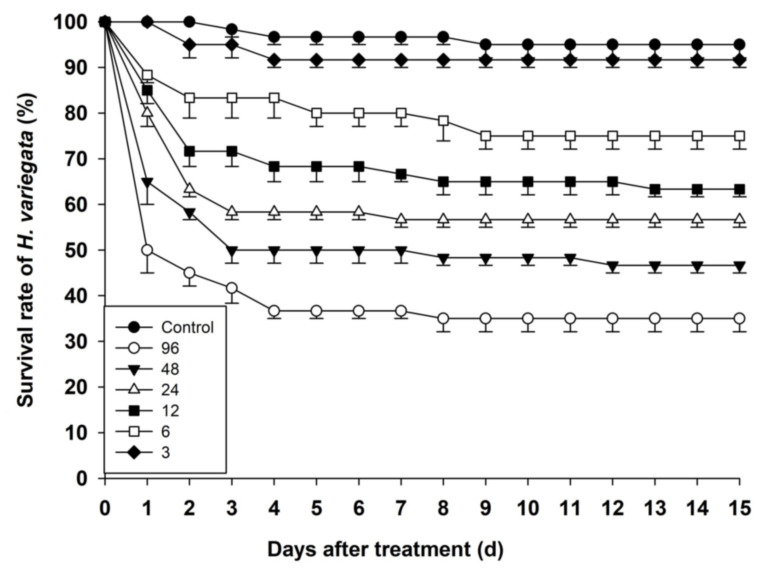
Survival rates of *H. variegata* larvae at six different doses (ng a.i. per insect) of sulfoxaflor during the 15 d observation period of a long-term toxicity test. Data are expressed as the mean values ± SE (standard error), *n*  =  3.

Figure 1 and Table 1 illustrate how sulfoxaflor affects the survival rate of *H. variegata*. Survival of *H. variegata* treated with sulfoxaflor at 9 and 48 ng a.i. per insect significantly declined compared to the control group. However, there were no statistically significant differences among 6, 12, and 24 ng a.i. per insect treatments. The mortality rates of *H. variegata* on the 15th day of the experiment were 5.00%, 8.33%, 25.00%, 36.67%, 43.33%, 53.33%, and 65.00%, in 0 (control), 3, 6, 12, 24, 48, and 96 ng a.i. per insect treatments, respectively. 

**Figure 2:**
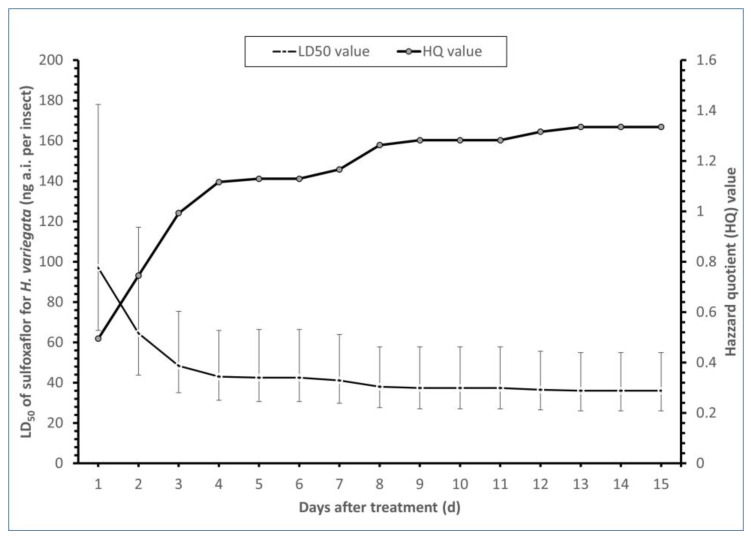
Estimated HQ and LD_50_ values of *H. variegata* in response to sulfoxaflor. Error bars correspond to the 95% confidence intervals (95% CI).

The estimated LD_50_ of sulfoxaflor for the 2nd instar *H. variegata* larvae 72 h after treatment was 48.35 ng ai per insect (95% confidence intervals 35.06–75.38 ng ai per insect), and it declined to 35.97 ng a.i. per insect 15 days after treatment (95% confidence intervals 26.06–54.88 ng ai per insect). The daily HQs for the second instar *H. variegata* larvae from day 1 to day 15 ranged from 0.5 to 1.33, all lower than 2, which is the limit of concern (Figure 2).

### 3.2. Influence of Sulfoxaflor on the Developmental Time, Female and Male Adult Longevity, and Female Pre-Oviposition Period of H. variegata

The growth period (2nd to 4th instar) for *H. variegata* larvae treated with sulfoxaflor lasted about seven to eight days, followed by pupation (four days). The larval growth period for the control group was significantly shorter than for the sulfoxaflor group (ANOVA, *p* < 0.05, NOER = 3 ng a.i. per insect). The pupal stage duration was significantly longer for the sulfoxaflor treated groups at doses above 12 ng a.i. per insect than for the controls (ANOVA, *p* < 0.05, NOER = 12 ng a.i. per insect). The APOP and the TPOP were significantly prolonged for the sulfoxaflor treated groups compared to the control group (ANOVA, *p* < 0.05, NOER = 12 and 3 ng a.i. per insect for APOP and TPOP, respectively). The longevities of female predator coccinellids exposed to 48 and 96 ng a.i. of sulfoxaflor per insect were 41.08 and 41.18 days, respectively, and they were significantly shorter compared to the control group (ANOVA, *p* < 0.05, NOER = 24 ng a.i. per insect). The male adult longevity was decreased as the doses increased, but it did not change significantly between the control and treatment groups (Table 2). 

### 3.3. Influence of Sulfoxaflor on Fecundity, Adult Weight, Sex Ratio, Total Effect, and IOBC Toxicity Categories of H. variegata

There were no statistically significant differences in the female proportion after exposure to sulfoxaflor (*p* = 0.800; NOER > 96 ng a.i. per insect). Sulfoxaflor 24–96 ng a.i. per insect significantly reduced the *H. variegata* adult weight (*p* < 0.05; NOER = 12 ng a.i. per insect). Compared to the control group, doses of 12–96 ng a.i. per insect significantly decreased the mean fecundity of females (*p* < 0.05; NOER = 6 ng a.i. per insect). The mean female fecundity decreased as the dose of sulfoxaflor increased (Table 2). 

### 3.4. Influence of Sulfoxaflor on Population Parameters and Population Projection of H. variegata

The sulfoxaflor treatments significantly reduced the net reproduction rate (*R_0_*), the finite rate of increase (*λ*), and the intrinsic rate of increase (*r*) values compared to the control (Table 3). The differences were significant at doses ranging 6–96 ng a.i. per insect. The mean generation time (*T*) was higher for all doses when *H. variegata* larvae were exposed to sulfoxaflor, compared to the control. 

**Figure 3:**
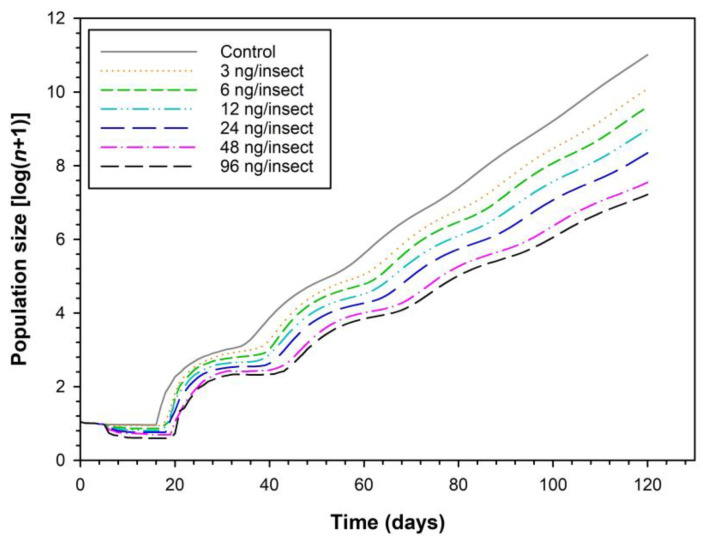
Population projection for *H. variegata* larvae exposed to different doses of sulfoxaflor. The estimated population size of *H. variegata* from an initial population in which 2nd instar larvae were treated with acetone (control) or 3, 6, 12, 24, 48, and 96 ng per insect of sulfoxaflor, respectively.

Figure 3 shows the projected population size of *H. variegata* larvae during 120 days from a given initial population and following different treatments. The population size of *H. variegata* after 120 days in the sulfoxaflor group projected to be 7.2 to 10.1-fold larger than the initial population, whereas, for the control group, the size was projected to reach 11.0-fold compared to the initial population. 

## 4. Discussion

Chemical insecticides have been the only used method to control aphids [3]. However, this had led to the development of insecticide resistance with noticeable examples those of *M. persicae* and *Aphis gossypii* Glover (Hemiptera Aphididae) (see [23]), which have developed resistance to many classes of insecticides and are ranked among the ten most resistant arthropods worldwide [24]. Strategies involving mitigation of resistance, i.e., rotation of insecticides with different MoA, along with protection and augmentation of natural control agents, are of primary importance. More precise IPM strategies are required to slow down or suppress insecticide resistance. The present study focused on the determination of toxicity and safety aspects of sulfoxaflor on *H. variegata,* an important aphid predator. 

We found that the LD_50_ of sulfoxaflor to *H. variegata* 15 days post treatment decreased from 97.03 to 35.97 ng a.i. per insect because of the cumulative mortality that originated from the toxic effect of sulfoxaflor. The same LD_50_ value pattern was found in bioassays with clothianidin [25], nitenpyram [26] and imidacloprid [27], and *C. septempunctata,* indicating that sulfoxaflor had a potential risk for *H. variegata*. Furthermore, three days after imidacloprid application, the fourth instar larvae of *H. variegata* showed LD_50_ values 15.11 ng a.i. per insect [8], showing that neonicotinoids, such as imidacloprid, may be more toxic and of higher risk for *H. variegata* than sulfoxaflor. The obtained HQ values for *H. variegata* were always below the safety threshold value of 2, demonstrating that sulfoxaflor was relatively safe for this aphid predator. Similarly, a HQ value below 2 was calculated for the 2nd instar larvae of *Harmonia axyridis* (Coleoptera: Coccinellidae) after sulfoxaflor exposure [28], or for the 2nd instar larvae of *C. septempunctata* after nitenpyram exposure [26]. Nevertheless, HQ values greater than 2 were calculated for various neonicotinoid insecticides, such us thiamethoxam for adults of *Serangium japonicum* (Coleoptera: Coccinellidae) [29] and clothianidin for larvae of *C. septempunctata* [25]. In the present study, sulfoxaflor at 3 ng a.i. per insect appeared to be harmless to *H. variegata* larvae (IOBC Class I). However, exposure to 6–96 ng a.i. per insect appeared to be slightly harmful to *H. variegata* larvae (IOBC Class II), which shows that sulfoxaflor is considered as relatively safe.

The total effect calculation is often used to assess toxicity due to different doses of insecticides on beneficial insects, such us predators and parasitoids [28]. In the present study, sulfoxaflor at ≤3 ng a.i per insect was harmless (IOBC Class 1). Interestingly, at application rates between 6 and 96 ng a.i. per insect, sulfoxaflor was slightly harmful (IOBC Class 2), which is the level of relative safety. Similarly, sulfoxaflor was found harmless (IOBC Class 1) for third instar larvae of *Chrysoperla carnea* (Neuroptera: Chrysopidae) [30], but it was moderately harmful (IOBC Class 3) for *Nesidiocoris tenuis* (Hemiptera: Miridae) [31] and highly harmful (IOBC Class 4) for the fourth instar larvae of *Adalia bipunctata* (Coleoptera: Coccinellidae) [28]. Sulfoxaflor was moderately harmful (IOBC Class 3) at an application rate of 180 g a.i. per hectare, and it was slightly harmful at ≤90 g a.i. per hectare for *H. axyridis* (Coleoptera: Coccinellidae) [28]. The variation amongst these studies could be attributed to differences in the examined predator species or in the used bioassay methods [32]. The *E* values found in the present study suggest that sulfoxaflor at doses ≥6 ng a.i. per insect could be classified as slightly harmful for *H. variegate* larvae. At a dose corresponding to twice the label rate (96 ng a.i. per insect), sulfoxaflor was shown to be slightly harmful. These results suggest that sulfoxaflor could reduce *H. variegata* efficiency in IPM programs. 

Sulfoxaflor doses ≥3 ng a.i. per insect significantly extended the duration of larval and pupal stages of *H. variegata*. Extended preadult developmental time has been found in many aphid predators, such as *H. axyridis* [28], *Chrysoperla rufilabris* (Neuroptera: Chrysopidae) [33], and *C. carnea* [34] after treatment with sulfoxaflor. These findings can be explained either by the antifeeding effect of sulfoxaflor on coccinellid predators [35] and the consequent reduced food intake or by the fact that affected larvae may use their metabolic energy for sulfoxaflor detoxification at expenses of their development and growth [10,32,36]. 

In the present study, sulfoxaflor, except for the pupal duration time, significantly extended APOP (NOER < 24 ng a.i. per insect) and TPOP (NOER < 3 ng a.i. per insect). These findings agree to those reported in previous studies, which examined the effects of sulfoxaflor on *H. axyridis* [28,37]. Sulfoxaflor reduced the fecundity (NOER = 12 ng a.i. per insect), fresh mass (NOER = 24 ng a.i. per insect), and the adult emergence rate of *H. variegata* (NOER = 6 ng a.i. per insect). These results may be related to sulfoxaflor MoA. Sulfoxaflor adversely affects predators’ neurosecretory system, leading to tremor and partial or complete paralysis, decreasing nervous activity, feeding efficiency, and predatory energy [28]. 

Population growth parameters, such as the net reproductive rate (*R*_0_), intrinsic rate of increase (*r*), mean generation time (*T*), and finite rate of increase (*λ*), can provide valuable information about *H. variegata* population dynamics. Treatment at doses ≥6  ng sulfoxaflor per insect reduced the *R*_0_, *λ,* and *r* of *H. variegata.* Reduction in those population growth parameters could be associated with decreased adult fresh mass, survival, fecundity, and longevity [9,10,11,28,32,36,38]. Population parameter reduction for sulfoxaflor treated groups probably underwent the physiological antifeeding effect caused by sulfoxaflor. In general, this agrees with our results, in which we showed that sulfoxaflor, even at low exposure doses, reduces not only fecundity, but also adult fresh mass, longevity, APOP, TPOP, and the main population parameters of *H. variegata*. 

The effect of sulfoxaflor at sublethal doses was assessed only by direct application, so the sulfoxaflor effect may be more pronounced when the predator is exposed to the maximum field concentration rate indirectly (consuming contaminated plant material and/or prey during foraging) [39]. In addition, the sublethal effects can adversely affect not only the treated parental generation, but also the progeny of the exposed coccinellids via transgenerational effects [40]. Negative effects of sulfoxaflor on the next generation have been reported by [39] for *C. septempunctata* and by [37] for *H. axyridis*. Overall, the decreased demographic parameters of *H. variegata* demonstrated that low dosage of sulfoxaflor may affect survival and reproduction in the next generation, resulting in reduced biological control efficacy provided by *H. axyridis* [39].

## 5. Conclusions

Understanding the effect of pesticides on aphid predators will assist the improvement of combination strategies and, consequently, the IPM program effectiveness. Sulfoxaflor adversely affected many *H. variegata* life table parameters. Taken as a whole, implementation of sulfoxaflor and *H. variegata* in IPM practices should be carefully employed. 

## Figures and Tables

**Table 1:** Influence of sulfoxaflor on the cumulative mortality for the pre-adult period, sex ratio, fresh mass, fecundity, total effect, and IOBC toxicity categories of the insecticide applied on 2nd instar *H. variegata* larvae. Within each column, treatments sharing the same superscript letter were not significantly different (ANOVA, Tukey’s HSD Test, *p* ≤ 0.05).

Treatment	Dose Used (ng a.i. per Insect)	Proportion of Female (%)	Fresh Mass of Adults (mg)	Cumulative Mortality (%)	Fecundity (Eggs/Female)	Total Effect (E *)	IOBC Toxicity Category *
Control	-	56.61 ± 8.36 ^a^	10.20 ± 0.25 ^a^	05.00 ± 2.88 ^e^	804.72 ± 56.99 ^a^	-	-
Sulfoxaflor	3	56.33 ± 0.78 ^a^	10.02 ± 0.27 ^ab^	08.33 ± 1.67 ^e^	575.61 ± 53.65 ^ab^	11.32	1
	6	48.89 ± 1.11 ^a^	09.47 ± 0.29 ^abc^	25.00 ± 2.89 ^d^	532.14 ± 44.12 ^ab^	30.39	2
	12	47.44 ± 1.28 ^a^	09.34 ± 0.34 ^abc^	36.67 ± 1.67 ^c^	486.61 ± 58.78 ^b^	45.29	2
	24	43.94 ± 4.01 ^a^	08.74 ± 0.30 ^bc^	43.33 ± 1.67 ^bc^	431.33 ± 66.21 ^b^	55.91	2
	48	47.04 ± 15.40 ^a^	08.44 ± 0.33 ^c^	53.33 ± 1.67 ^b^	435.23 ± 65.73 ^b^	61.00	2
	96	52.38 ± 4.12 ^a^	08.31 ± 0.35 ^c^	65.00 ± 2.89 ^a^	392.00 ± 72.90 ^b^	73.72	2

* The IOBC toxicity categories for laboratory experiments are in accordance with the total effects caused by insecticides: (1) harmless (E < 30%); (2) slightly harmful (30% ≤ E ≤ 79%); (3) moderately harmful (80% ≤ E ≤ 99%); and (4) highly harmful (E > 99%).

**Table 2:** Development time of *H. variegata* when 2nd-instar larvae were treated with sulfoxaflor. Within each column, treatments sharing the same superscript letter were not significantly different (ANOVA, Tukey’s HSD Test, *p* ≤ 0.05).

Treatment	Dose Used (ng a.i. per Insect)	Duration of Different Life Stages (d)								
		2nd Instar	3rd Instar	4th Instar	2nd to 4th Instar	Pupae	Female Adult Longevity (d)	APOP^a^	TPOP^b^	Male Adult Longevity (d)
Control	-	1.65 ± 0.064 ^b^	1.79 ± 0.070 ^c^	3.46 ± 0.087 ^b^	6.89 ± 0.111 ^b^	3.70 ± 0.075 ^b^	60.31 ± 2.84 ^a^	2.03 ± 0.18 ^d^	17.72 ± 0.29 ^d^	43.84 ± 3.03 ^a^
Sulfoxaflor	3	2.05 ± 0.048 ^a^	1.82 ± 0.059 ^bc^	3.89 ± 0.106 ^ab^	7.76 ± 0.104 ^a^	4.07 ± 0.100 ^ab^	46.94 ± 2.82 ^ab^	2.32 ± 0.21 ^cd^	19.29 ± 0.29 ^c^	41.04 ± 3.09 ^a^
	6	2.04 ± 0.044 ^a^	2.07 ± 0.074 ^abc^	3.89 ± 0.079 ^ab^	8.00 ± 0.119 ^a^	4.09 ± 0.083 ^ab^	45.23 ± 4.29 ^ab^	2.46 ± 0.24 ^cd^	19.77 ± 0.35 ^bc^	38.83 ± 3.16 ^a^
	12	1.97 ± 0.070 a	2.13 ± 0.086 ^ab^	3.87 ± 0.094 ^ab^	7.97 ± 0.144 ^a^	4.08 ± 0.087 ^ab^	44.44 ± 4.32 ^ab^	3.06 ± 0.36 ^bcd^	20.44 ±0.38 ^abc^	34.95 ± 3.38 ^a^
	24	1.88 ± 0.082 ^ab^	2.06 ± 0.072 ^abc^	3.74 ± 0.088 ^ab^	7.68 ± 0.132 ^a^	4.12 ± 0.101 ^a^	43.73 ± 4.32 ^ab^	3.47 ± 0.31 ^abc^	20.53 ± 0.42 ^abc^	36.47 ± 3.47 ^a^
	48	1.68 ± 0.090 ^b^	2.07 ± 0.102 ^abc^	3.86 ± 0.112 ^ab^	7.61 ± 0.165 ^a^	4.21 ± 0.079 ^a^	41.08 ± 6.68 ^b^	4.31 ± 0.54 ^ab^	21.62 ± 0.45 ^a^	34.27 ± 3.91 ^a^
	96	1.62 ± 0.129 ^b^	2.19 ± 0.088 ^a^	3.95 ± 0.129 ^a^	7.76 ± 0.217 ^a^	4.14 ± 0.104 ^a^	41.18 ± 5.19 ^b^	4.55 ± 0.68 ^a^	21.54 ± 0.49 ^ab^	27.80 ± 4.79 ^a^

^a^ Adult pre-oviposition period. ^b^ Total pre-oviposition period.

**Table 3:** Estimates of life table parameters of *H. variegata* when 2nd-instar larvae were treated with sulfoxaflor. Within each column, treatments sharing the same superscript letter were not significantly different on the paired bootstrap test at the 5% significance level.

Treatment	Dose Used (ng a.i. per Insect)	Life Table Parameters			
		Net Reproduction Rate (*R_0_*) (Female Female^−1^)	Intrinsic Rate of Increase (*r*) (Female Female^−1^d^−1^)	Finite Rate of Increase (*λ*) (Female Female^−1^d^−1^)	Mean Generation Time (*T*) (d)
Control	-	362.69 ± 53.94 ^a^	0.2053 ± 0.0086 ^a^	1.2279 ± 0.0105 ^a^	28.71 ± 0.74 ^b^
Sulfoxaflor	3	251.32 ± 40.99 ^ab^	0.1869 ± 0.0073 ^ab^	1.2055 ± 0.0088 ^ab^	29.57 ± 0.56 ^a^
	6	164.89 ± 31.67 ^bc^	0.1774 ± 0.0083 ^bc^	1.1941 ± 0.0099 ^bc^	28.78 ± 0.68 ^b^
	12	123.37 ± 29.03 ^cd^	0.1658 ± 0.0099 ^bcd^	1.1804 ± 0.0117 ^bcd^	29.04 ± 0.66 ^b^
	24	91.13 ± 24.90 ^cd^	0.1542 ± 0.0113 ^cd^	1.1667 ± 0.0131 ^cd^	29.26 ± 0.79 ^a^
	48	79.69 ± 23.76 ^d^	0.1400 ± 0.0105 ^d^	1.1503 ± 0.0120 ^d^	31.27 ± 0.68 ^a^
	96	60.73 ± 20.03 ^d^	0.1339 ± 0.0141 ^d^	1.1433 ± 0.0160 ^d^	30.67 ± 1.45 ^ab^

## Data Availability

The data presented in this study are available on request from the corresponding author.

## Supplementary Materials

The following supporting information can be downloaded at: https://www.mdpi.com/article/10.3390/toxics11060533/s1, “References [41,42,43,44,45] are cited in the Supplementary Materials”.
