# Supplementary material for: Toxicity and Influence of Sublethal Exposure to Sulfoxaflor on the Aphidophagous Predator Hippodamia variegata (Coleoptera: Coccinellidae)
Source: Toxics. 2023 Jun 14;11(6):533. doi: 10.3390/toxics11060533 (PMC10305232; doi:10.3390/toxics11060533)
Supplement: Supplementary file 1 [file toxics-11-00533-s001.zip › toxics-2377749-supplementary.pdf]

Supplementary Materials: Toxicity and Influence of Sublethal Exposure to Sulfoxaflor on the Aphidophagous Predator *Hippodamia variegata* (Coleoptera: Coccinellidae)

Panagiotis J. Skouras [1,2,*], Eirini Karanastasi [3], Vasilis Demopoulos [2], Marina Mprokaki [1], George J. Stathas [1] and John T. Margaritopoulos [4]

1 Laboratory of Agricultural Entomology and Zoology, Department of Agricultur, Kalamata Campus, University of the Peloponnese, 24100 Antikalamos, Greece; marina.brokaki.97@gmail.com (M.M.); g.stathas@uop.gr (G.S.)
2 Laboratory of Plant Protection, Department of Agricultur, Kalamata Campus, University of the Peloponnese, 24100 Antikalamos, Greece; v.dimopoulos@go.uop.gr
3 Plant Protection Laboratory, Department of Agriculture, University of Patras, 30200 Messolonghi, Greece; ekaranastasi@upatras.gr
4 Department of Plant Protection, Institute of Industrial and Fodder Crops, Hellenic Agricultural Organization "DEMETER", *38*334 Volos, Greece; johnmargaritopoulos@elgo.com
* Correspondence: author: p.skouras@go.uop.gr or pskouras@windowslive.com; Tel.: +30-27210-45277; Fax: +30-27210-45234;

Population parameters

The basic life table and population parameters were calculated using the program TWOSEX-MS Chart [41].

The net reproductive rate (R_0) is calculated as: $R_0 = \sum_{x=0}^{\infty} l_x m_x$

Intrinsic rate of increase (r): $\sum_{x=0}^{\infty} e^{-r(x+1)} l_x m_x = 1$

Mean generation time (T): $T = \frac{\ln R_0}{r}$

Finite rate of increase (λ): $\lambda = e^r$

were calculated according to Chi and Liu (1985) [42] and Chi 1988 [43]. The means ± standard error of the life table parameters were estimated by the bootstrap procedure with 200.000 replicates and the means for each treatment were compared using the paired bootstrap test (*P<0.05*) [44].

Population projection

The life table data for one generation following different treatments of sulfoxaflor were used to project the population using the free TIMING-MSChart software program [45]. The software program waqs obtained from http://140.120.197.173/Ecology/prod02.htm. The population size was projected over 120 days from an initial population.

References

41. Chi, H. TWOSEX-MSChart: A Computer Program for the Age-Stage, Two-Sex Life Table Analysis. National Chung Hsing University, Taichung, Taiwan. (2021). Available online: http://140.120.197.173/Ecology/ (accessed on 1 November 2022).
42. Chi, H.; Liu, H. Two new methods for the study of insect population ecology. *Bull. Inst. Zool. Acad. Sin.* **1985**, *24*, 225–240.
43. Chi, H. Life-Table Analysis Incorporating Both Sexes and Variable Development Rates Among Individuals. *Environ. Entomol*. **1988**, *17*, 26–34.
44. Akköprü, E.P.; Atlıhan, R.; Okut, H.; Chi, H. Demographic assessment of plant cultivar resistance to insect pests: A case study of the dusky-veined walnut aphid (Hemiptera: Callaphididae) on five walnut cultivars. *J. Econ. Entomol*. **2015**, *108*, 378–387.
45. Chi, H. *TIMING-MSChart: A Computer Program for the Population Projection Based on Age-Stage, Two-Sex Life Table*; National Chung Hsing University: Taichung, Taiwan, 2020.
